# Knowledge of Chikungunya Fever Among Medical Students of Jinnah Sindh Medical University, Karachi, Pakistan

**DOI:** 10.7759/cureus.3179

**Published:** 2018-08-21

**Authors:** Shafaq Jawed, Shajeeya Khaliq, Masooma Abid, Javeryah R Shaikh, Osama Salam, Zain Abid

**Affiliations:** 1 Medicine, Jinnah Sindh Medical University, Karachi, PAK; 2 Sindh Medical College, Jinnah Sindh Medical University (SMC), Karachi, PAK; 3 MBBS Student, Jinnah Medical and Dental College, Karachi, PAK; 4 Miscellaneous, Jinnah Sindh Medical University (SMC), Karachi, PAK; 5 Internal Medicine, Dow University of Health Sciences (DUHS), Sindh Medical College, Karachi, PAK; 6 Department of Oncology, Jinnah Postgraduate Medical Center, Karachi, PAK

**Keywords:** chikungunya, virus, medical students, mosquito, viral fever, fever, public awareness, analgesics

## Abstract

Objective

Chikungunya is a viral disease characterized by severe arthralgia, fever, rash, muscle pain, and neurological symptoms. Warm and humid weather, poor sanitary conditions, and improper water storage and disposal can lead to an uncontrolled outbreak of the Chikungunya virus in South Asia. Because a vaccine against the Chikungunya virus has not yet been developed, we must rely on appropriate awareness and suitable preventive measures to prevent its spread. A review of the literature shows that knowledge of the Chikungunya virus among medical students in Karachi is scarce. Because medical students are future medical practitioners, they should be adequately aware of this growing issue.

Materials and methods

We performed a single-center, cross-sectional study at the Jinnah Sindh Medical University in Karachi, Pakistan, in which 200 students were assessed on their knowledge of the Chikungunya virus and fever via a structured questionnaire.

Results

A total of 200 students participated in the study. The mean age of the study participants was 20 ± 1 years. Only 50% of the study participants had an adequate knowledge score related to the Chikungunya virus and fever. We found that 43% had an average knowledge, and 7% had poor knowledge. The study also revealed a strong association between year of study and knowledge score (p=0.003); those in the higher age group had more knowledge (p=0.014).

Conclusion

A low percentage of medical students have sufficient knowledge about Chikungunya virus and fever, which is alarming because Pakistan has recently faced a severe epidemic of Chikungunya virus fever and is a country prone to further outbreaks. Multiple training programs and lectures are necessary to prepare and educate medical students about both basic and clinical knowledge of Chikungunya.

## Introduction

World Health Organization (WHO) defines Chikungunya fever as a viral disease characterized by severe arthralgia, fever, rash, and muscle pain. Various cases involving neurological symptoms, such as encephalitis, myelopathy, and peripheral neuropathy have also been reported [1]. Chikungunya virus is an arthropod-borne alphavirus belonging to the Togavirus family. Its genomic makeup consists of a positive ribonucleic acid strand with seven proteins (four non-structural and three structural proteins) [2].

The name “Chikungunya” originates from the Makonde language spoken in southern Tanzania and means “that which bends joints” [2]. The translation refers to the flexed posture of patients suffering from Chikungunya fever to relieve joint pains [3]. The first encounter of Chikungunya virus was in the Newala district of Tanzania in 1953; people of variable ages were affected and the virus spread rapidly to involve 49 of the 62 localities. [4]. This discovery was soon followed by outbreaks in several parts of Africa, including Nigeria, Malawi, Uganda, and Burundi. The actual spread of the Chikungunya virus in South Asia occurred in 2005. Due to similarities in clinical symptoms, this was initially mistaken for a dengue virus outbreak, which caused misdiagnoses and an underestimation of the actual number of reported cases [5].

Theories vary regarding the emergence of Chikungunya virus in the South Asian subcontinent. Some researchers suggest it was the result of an outbreak in Kenya, which resulted in a seropositivity index of 70%. Other researchers suspect Uganda was the route of spread into South Asia [6]. The disease was reported again, long after its first encounter, in which many South Asian regions were affected, including regions of India and Pakistan where seroprevalence rates were 9.91% (in 2010, in the national capital region of India) and 12.6% (in 2016 in Mumbai, India) [7]. The outbreak in India was followed by a massive spread in Karachi, Pakistan, in which approximately 30,000 people were disease-ridden. However, only 800 of these cases were reported by the WHO [8]. After the major recurrence in the Indian Ocean in 2005, the Chikungunya virus has posed a major threat to the world’s population. Warm and humid weather, poor sanitary conditions, and improper water storage and disposal are factors that facilitate its uncontrolled outbreak in South Asia [9-10]. Aedes aegypti and Aedes albopictus are the two most significant species of Aedes mosquitoes responsible for the spread of the Chikungunya virus worldwide. However, in South Asia, the use of plastic bags and tires as water storage have proved to be a major factor in increased disease spillover and improved survival of Aedes albopictus and Aedes aegypti, respectively [7].

The re-emergence, rapid spread, and travel epidemiological outbreak of the Chikungunya virus have posed a serious challenge to the government and healthcare professionals in preventing a future outbreak. Furthermore, the vaccine for its prevention is currently in progress. Thus, appropriate awareness and suitable preventive measures are the only techniques available to prevent its spread. Reviews of the available literature show that knowledge regarding the Chikungunya virus among medical students in Karachi is very scarce. Medical students are future practitioners, and they should be adequately aware of the Chikungunya virus. Previously, few studies have been conducted in Pakistan for assessing the knowledge of viral fevers in medical students. However, no previous study regarding the knowledge of Chikungunya virus in medical students has been reported. Given this disease is currently in an endemic phase in Pakistan, with Karachi reporting 803 cases alone [11], it is crucial for the medical students of Karachi, Pakistan, to have sufficient information about the modalities, management, and preventive measures for the Chikungunya virus. Therefore, this study will assess the knowledge gap related to Chikungunya and provide us with information to help estimate the extent of efforts required to fill that knowledge gap.

## Materials and methods

We conducted a cross-sectional study at the Jinnah Sindh Medical University in Karachi, Pakistan. A multistage, random, stratified sampling technique was implemented to select participants for this study. The sample size was calculated using the WHO calculator, and the confidence interval was set to 95%. The minimum sample size was 200 participants.

Our inclusion criteria were adult men and women aged 18 to 23 years enrolled in Sindh Medical College who agreed to participate. Each participant provided written informed consent after learning the details of their role in the study and its aim and objectives. Data were collected using a self-structured validated questionnaire. Questions were designed based on previously published studies regarding the knowledge and attitude of virus epidemics among institutions. Google Scholar and PubMed were the principal electronic bibliographic sources for these published articles. The questionnaire was composed of multiple-choice questions (MCQs) divided into three parts; the first part inquired about their demographics, year of program, and source of knowledge about the virus. The second part consisted of 10 questions each on the following topics: causative agent, the pattern of transmission, the management of patients affected by Chikungunya, knowledge of vaccination, and prevention. In the last part, participants were asked if Pakistan has enough resources to control and manage the epidemic of Chikungunya in their region.

Data analysis was done using IBM Statistical Package for the Social Sciences (SPSS) Statistics for Windows, Version 21.0. (IBM Corp., Armonk, NY). Each MCQ had only one correct answer choice. The participants were assessed by receiving one point for the correct answer and zero for all incorrect choices. The knowledge scores were computed based on the total number of correct answers. Ten was the highest score achieved by any participant. A score below three indicates poor knowledge, a score from three to five indicates average knowledge, five to eight was satisfactory, and scores above eight indicate adequate knowledge. A p-value of <0.5 was considered significant. Descriptive statistics of independent and dependent variables were presented as mean, standard deviation, or frequency percentages. The questionnaire was validated using Cronbach's alpha (0.801).

## Results

A total of 200 students participated in the study. The mean age of the participants was 20±1 years. Most respondents belonged to the second year of their study (51%), followed by first-year students (19%), third-year students (14%), fourth-year students (13.5%), and, lastly, fifth-year students (2.5%). The most common source of information about Chikungunya virus and fever was television (41%; n = 82) followed by the Internet (24%; n = 48; Table 1).

**Table 1:** Sociodemographic characteristics

		Frequency	Percentage	Valid Percentage
Age (Years) (Mean=20±1)	18-19	64	32.0	32.2
	20-21	104	52.0	52.3
	22-23	31	15.5	15.6
	Total	199	99.5	100.0
	Missing	1	.5	
Year of Education	1^st^	38	19.0	19.0
	2^nd^	102	51.0	51.0
	3^rd^	28	14.0	14.0
	4^th^	27	13.5	13.5
	5^th^	5	2.5	2.5
Source	Television	82	41.0	41.0
	Internet/Books/Newspaper	48	24.0	24.0
	Family/Friends	41	20.5	20.5
	Awareness Programs	16	8.0	8.0
	Others	13	6.5	6.5

Nearly all participants (95.5%) marked the virus as the cause of Chikungunya fever. While 93.5% of the participants identified fever and joint pain as the main symptom of the fever, only 66.5% knew serological assays are the definitive diagnostic test for this fever. The mode of transmission and vector association was marked correctly by 79% and 78.5% students, respectively. Regarding the human transmission of the disease, 67% reported it was not possible while 33% believed that it was possible. About 41% of students believed that vaccines against Chikungunya virus are available while 59% knew the vaccine was not available. Fifty-nine percent of the medical students chose analgesics as the most effective management option for the fever. A majority (61.5%) of them marked mosquitoes’ repellent as a preventive measure. Interestingly, only 38% of participants were aware that a hot and humid climate is the ideal environmental factor for the spread of the fever (Table 2).

**Table 2:** Knowledge of Chikungunya virus

		Frequency	Percentage	Valid Percentage
Awareness of Name	No	1	.5	.5
	Yes	199	99.5	99.5
Cause	Chicken, Dust, Hot climate	9	4.5	4.5
	Virus (Correct)	191	95.5	95.5
Symptoms	Bloody stools, Chest pain, Don't know	13	6.5	6.5
	Fever and Joint Pain (Correct)	187	93.5	93.5
Diagnosis	Blood Tests, Urinalysis, Don't Know	67	33.5	33.5
	Serological Test (Correct)	133	66.5	66.5
Mode of Transmission	Airborne, Food/Water, Don't Know	42	21.0	21.0
	Vector (Correct)	158	79.0	79.0
Vector Types	Lice, Ticks, Don't Know	43	21.5	21.5
	Mosquitoes (Correct)	157	78.5	78.5
Possible Human Transmission	Yes	66	33.0	33.0
	No (Correct)	134	67.0	67.0
Vaccine	Yes	82	41.0	41.0
	No (Correct)	118	59.0	59.0
Effective Management	Antibiotics, Blood thinners, Don't Know	82	41.0	41.0
	Analgesics (Correct)	118	59.0	59.0
Adequate Climate for the Virus	Any Other	124	62.0	62.0
	Hot and Humid (Correct)	76	38.0	38.0
Preventive Management	Don't know	77	38.5	38.5
	Mosquito Repellent (Correct)	123	61.5	61.5

Half of the participants had adequate knowledge regarding Chikungunya virus and fever. The percentage of individuals with average knowledge was 43%, and 7% had a poor knowledge level (Table 3).

**Table 3:** Knowledge score of participants

	Frequency	Percentage	Valid Percentage
Poor (score of <3)	13	6.5	6.5
Average (score of 4-7)	86	43.0	43.2
Adequate (score of 8-10)	100	50.0	50.3
Total	199	99.5	100.0
Missing	1	.5	
Final Total	200	100.0	

Pearson’s chi-squared test revealed a strong association between year of study and knowledge score (p=0.003) as represented in Table 4. The older age group (those aged 22 to 24 years) had a greater tendency of achieving an adequate score (77%) as compared to younger groups (those under 20 years old), where only 40.6% of participants were able to reach an adequate score (Table 5). The differences were statistically significant (p=0.014). About 79% (n=158) of participants indicated Pakistan does not have a sufficient strategy or level of resources to manage and control the spread of Chikungunya fever (Table 6).

**Table 4:** Correlation between academic year and knowledge level of Chikungunya virus

		Score			Total	P-Value
		Poor	Average	Adequate		
Year of Education	1^st^	4	21	13	38	0.003
	2^nd^	9	49	43	101	
	3^rd^	0	10	18	28	
	4^th^	0	6	21	27	
	5^th^	0	0	5	5	
Total		13	86	100	199	

**Table 5:** Correlation between age and knowledge level of Chikungunya virus

		Score			Total	P-Value
		Poor	Average	Adequate		
Age Ranges	18-19 Years	6	32	26	64	0.014
	20-21 Years	7	47	49	103	
	22-23 Years	0	7	24	31	
Total		13	86	99	198

**Table 6:** Does Pakistan have effective management?

	Frequency	Percentage	Valid Percentage
Yes	42	21.0	21.0
No	158	79.0	79.0
Total	200	100.0	100.0

## Discussion

Pakistan faced a major Chikungunya outbreak in November 2016, involving 4,000 cases, according to the reports [8]. Pakistan is a limited resource country where the adoption of preventive measures by the population and the efficient use of resources by healthcare staff are the preferred ways to combat an epidemic. Medical students are future health care practitioners, and their knowledge of ongoing health hazards to the population is critical. Our study reveals that only half of the participating medical students had adequate knowledge of Chikungunya fever. This is a major concern as medical students participate in various care provision activities during their hospital rounds and are exposed to patients suffering from epidemic viruses quite often. Our study reveals that age and year of study have a direct correlation with an adequate knowledge score; this contradicts a previous study reporting that private clinicians had low knowledge scores in comparison to postgraduate trainees and medical officers [12].

A large percentage of participants in our study were not aware of the management protocol regarding the Chikungunya virus and were unaware of the self-resolving behavior of Chikungunya fever. This finding is similar to that of a study conducted in India where health practitioners believed that Ayurveda or homeopathic treatment was the most efficient way of combating Chikungunya virus-induced fever [13].

The reasons behind this gap in knowledge are yet to be evaluated, and the lack of awareness programs and campaigns might be a causative factor. Much better results were obtained in a similar study on link workers of Urban Health Centers in Ahmedabad, India. This study involved a pre- and post-training evaluation of Chikungunya knowledge and concluded there was a significant improvement in the awareness of link workers after a 14-day education training [14].

It is necessary for medical students to be aware of basic prevention measures for viral epidemics, as they are a major source of counseling for the population to implement these steps. Our study showed that only 61.5% of the medical students were aware of mosquito repellents as a preventive measure against the Chikungunya virus. A study conducted on the medical students of Cambodia showed improved results; 97% of those medical students had knowledge of Chikungunya virus prevention strategies [15]. The cause of such a high level of knowledge might be the massive spread of Chikungunya in Cambodia where the epidemic was much more severe than what had occurred in Pakistan.

The vaccine against Chikungunya is the most cost-effective measure. However, it is only in phase two of its clinical trials. Antivirals have shown promising outcomes in vitro [16].

Only 59% of participants were aware of the current unavailability of the vaccine against Chikungunya. Risk perception is an important predictor of the attitude of an individual towards disease severity. These low numbers might not have a short-term impact, but they would have unwanted long-term outcomes, as these figures show a large number of medical students are unaware of the seriousness of the Chikungunya virus epidemic in Pakistan. Many respondents in our study had a despairing attitude regarding Pakistan’s stance toward combating the Chikungunya virus. This result is consistent with two studies conducted in India where the population attributed corruption and negligence as key factors of the ineffective mitigation of the spread of the Chikungunya virus [17-18].

This is the first-ever study done in Pakistan assessing Chikungunya virus knowledge among medical students. The study has some limitations. Firstly, this is a single-center study, and it might not be able to represent the knowledge and perception of this virus in the large population of medical students in Pakistan. Secondly, due to the application of random selection criteria, a selection bias may occur. Therefore, we took a large sample to mitigate the degree of bias.

## Conclusions

A low percentage of medical students have sufficient knowledge of the Chikungunya virus and the associated fever. This is alarming because Pakistan has recently faced a severe epidemic of Chikungunya virus fever and is prone to yet another outbreak. Medical students, as future health practitioners, require adequate knowledge of the Chikungunya virus to fulfill their roles as health care professionals during epidemics. Multiple training programs and lectures are needed to prepare and educate medical students on the basic and clinical aspects of Chikungunya.
